# Recent Advances of Microneedles and Their Application in Disease Treatment

**DOI:** 10.3390/ijms23052401

**Published:** 2022-02-22

**Authors:** Wenjing Zhang, Wei Zhang, Cairong Li, Jianhua Zhang, Ling Qin, Yuxiao Lai

**Affiliations:** 1 Center for Translational Medicine Research and Development, Institute of Biomedical and Health Engineering, Shenzhen Institute of Advanced Technology, Chinese Academy of Sciences, Shenzhen 518055, China; wj.zhang2@siat.ac.cn (W.Z.); zhang.wei@siat.ac.cn (W.Z.); cr.li@siat.ac.cn (C.L.); jh.zhang2@siat.ac.cn (J.Z.); qin@ort.cuhk.edu.hk (L.Q.); 2 University of Chinese Academy of Sciences, Beijing 100049, China; 3 Musculoskeletal Research Laboratory, Department of Orthopedics and Traumatology, The Chinese University of Hong Kong, Hong Kong 999077, China; 4 CAS-HK Joint Lab of Biomaterials, Shenzhen 518055, China; 5 Key Laboratory of Health Informatics, Shenzhen Institute of Advanced Technology, Chinese Academy of Sciences, Shenzhen 518055, China

**Keywords:** microneedles, drug delivery, nanotechnology, disease treatment

## Abstract

For decades, scientists have been doing a lot of research and exploration to find effective long-term analgesic and/or disease-modifying treatments. Microneedles (MNs) are a simple, effective, and painless transdermal drug delivery technology that has emerged in recent years, and exhibits great promise for realizing intelligent drug delivery. With the development of materials science and fabrication technology, the MN transdermal drug delivery technology has been applied and popularized in more and more fields, including chronic illnesses such as arthritis or diabetes, cancer, dermatocosmetology, family planning, and epidemic disease prevention, and has made fruitful achievements. This paper mainly reviews the latest research status of MNs and their fabrication methodology, and summarizes the application of MNs in the treatment of various diseases, as well as the potential to use nanotechnology to develop more intelligent MNs-based drug delivery systems.

## 1. Introduction

Syringes and hypodermic needles have been used to deliver drugs to patients for more than 160 years and are still the most routine and effective means [1]. However, there are some obvious limitations of this traditional syringe injection or needle delivery method in clinical application. First of all, there is obvious pain resulting from needle insertion, and a large proportion of patients, especially children, show needle phobia and poor compliance, causing patients to psychologically reject the treatment, seriously affecting the effectiveness of the drugs. Secondly, needle reuse errors may lead to risk of infection with blood-borne pathogens, posing an incalculable risk to patients. In addition, drug administration requires practitioners with a foundation of professional training to perform drug administration, which is very inconvenient, especially for patients requiring frequent drug administration. Therefore, the cost and risk should be taken into consideration. Moreover, the needles and syringes used by patients are mostly disposable medical supplies, resulting in the production of a lot of sharp waste [2]. In addition to percutaneous administration, there are also obvious problems with other conventional administration methods. For example, oral administration cannot be used for many drugs due to the limitation of enzyme metabolism in the gastrointestinal tract and liver, leading to low bioavailability of drugs [3]. Medicated baths are a traditional percutaneous administration method, but due to skin obstruction, the medicinal effect is greatly degraded, while the medication is also inconvenient. Topical ointment administration involves the penetration of the drug through the skin into the body, which is limited to lipophilic small molecule drugs [2,4]. Hypodermic injection is still an irreplaceable method of drug delivery, as it can enable the drug to reach the lesion site more quickly and effectively. Therefore, developing hypodermic delivery methods that can alleviate pain and infection is particularly important for effective drug delivery.

The skin is the largest organ, and is a strong biological barrier, preventing infectious diseases and harmful substances from entering the body. However, the skin is also a major barrier to the effective delivery of drugs to lesions via percutaneous administration, while meanwhile also being susceptible to pain [5]. The skin consists of three morphologically distinct layers: the cuticle, the living epidermis, and the dermis [6]. The skin acts as a barrier to drug absorption largely by means of the outermost stratum corneum (10–20 μm) [2,7,8,9]. The protective function of the skin is mainly provided by the stratum corneum. The stratum corneum consists of dead keratinocytes and intercellular matrix (commonly referred to as brick and mortar, respectively). The latter is composed primarily of cholesterol, triglycerides, and ceramides, forming a dense structure with lipophilic properties (1.4 g/cm^3^). Due to the dense structure, it is almost impossible for drugs with molecular weight >500 Da and Log P in the 1–3 range to penetrate the cuticle [10]. Drugs with low molecular weight (<500 Da), high lipophilicity, and requiring doses as low as a milligram are allowed to pass through the cuticle [9,11]. Below the cuticle is a living epidermis (50–100 μm), which is responsible for the formation of the cuticle. The living epidermis is composed of different layers—basal layer, spinous layer, and granular layer from inside to outside, respectively—and without neural networks [6,12,13]. The dermis (1–2 mm) includes a wide range of nerve endings and blood vessels, so it can feel pain [13].

Microneedles (MNs), as the name implies, are a drug delivery tool with a pointer size in the micron scale, and consists of an MN patch in the form of many MN arrays with a needle length of 25–1000 μm that can only penetrate the active cuticle and live epidermis without reaching the nerve endings and blood vessels. Because of the microscopic size of MNs, the use of MNs does not stimulate nerves in the dermis or damage blood vessels, and thus patients do not feel intense pain during the process [14,15]. MNs could also overcome patients’ fear of needles, and avoid the liver and adverse metabolic and gastrointestinal side effects of oral administration, while penetrating the barrier of the cuticle without producing obvious pain. In addition, through the unique material selection and design, it can achieve the sustained long release of drugs under specific conditions [16,17,18,19]. In this review, we firstly introduce the background and the typical variety of MN drug delivery methods, including solid MNs, coated MNs, hollow MNs, degradable or soluble MNs, swelling MNs and porous MNs. Then we summarize the typical MN fabrication techniques and recent progress. However, in real application, the complete process route for making target MNs usually requires a combination of multiple techniques. Next, we review the latest research status of MNs and summarize the application of MNs in the treatment of various diseases, including chronic illnesses such as arthritis, diabetes, cancer, dermatology, cosmetics, family planning, and epidemic disease prevention. Finally, this paper gives an overview of the functional design of MN drug delivery systems using nano-engineering technology, which could be used to realize intelligent control of drug release amount, release kinetics, or stimuli-responsive delivery. In addition, we also present a research prospect for combining nanotechnology with MNs technology to achieve intelligent drug delivery.

## 2. Types of MNs

**Figure 1:**
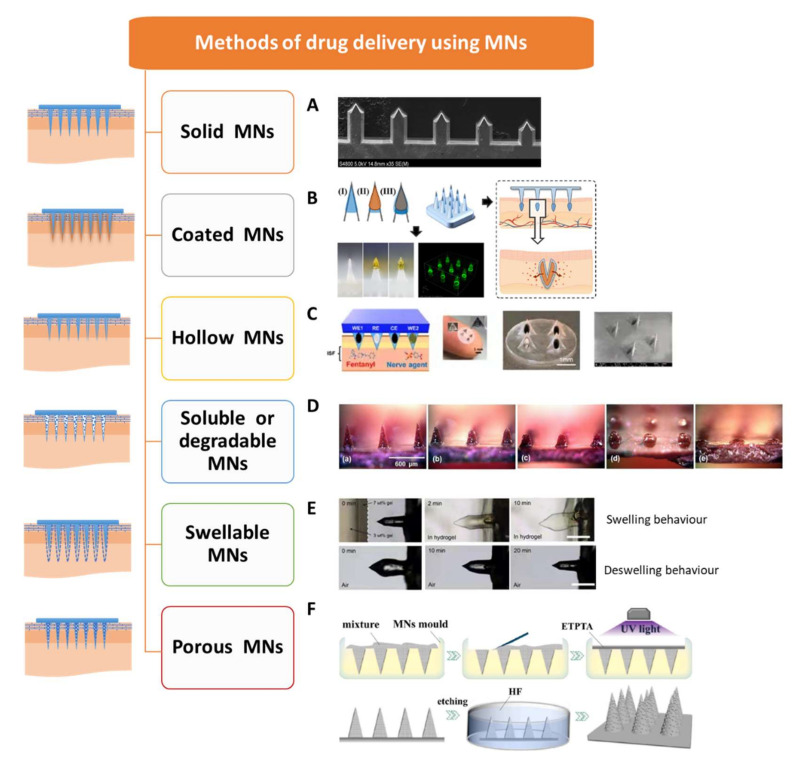
The typical variety of drug delivery methods using MNs. (**A**) Solid MNs. Adapted with permission from Ref. [22]. (**B**) Coated MNs. Adapted from Ref. [23]. (**C**) Hollow MNs. Adapted with permission from Ref. [24]. (**D**) Degradable or soluble MNs (a) before insertion, and (b) 10 s, (c) 1 min, (d) 15 min, and (e) 1 h after insertion into pig cadaver skin. Adapted with permission from Ref. [25]. (**E**) Swelling MNs. Adapted with permission from Ref. [26]. (**F**) Porous MNs. Adapted with permission from Ref. [27].

The development of a safe, fast, effective, and convenient drug delivery method is the main purpose of MNs research. According to different drug delivery methods, MNs can be divided into solid MNs, coated MNs, hollow MNs, degradable or soluble MNs, swelling MNs, and porous MNs [2,20,21]. Figure 1 shows the typical variety of drug delivery methods that use MNs.

### 2.1. Solid MNs

Solid MNs are made of silicon or metal without carrying drugs, which can be used for skin pretreatment. The MNs are removed after the punctured epidermis forms microchannels, and the drug preparation is applied to the puncture site and then diffuses into the body through the channels [22,28]. The microchannels gradually shrink as the skin heals over several hours until they close completely. This kind of MN administration process is divided into two tedious steps, and the number of drugs entering the body cannot be accurately measured. Recently, Howells et al. [22] presented simple single wet etch step fabricated solid MNs with different geometries to determine the optimal MN length and width for effective penetration and minimally invasive drug delivery (Figure 1A). In addition, silicon or metal materials have good mechanical force, but not enough bending force, so there will be a high risk of “breaking” during insertion, resulting in residual needle bodies being left behind.

### 2.2. Coated MNs

Coated MNs are surface drug-loaded MNs. The drug is attached to the surface of the MNs by dipping, spraying, or coating. The drug dissolves when the needle is inserted into the skin and is absorbed by the body. This method is mainly applicable to water-soluble drugs, which have the advantages of rapid drug release, high use rate, and easy dose control, as shown in Figure 1B [23]. However, the amount of drug-coated on the MNs is usually small, and the application scope is limited to just those cases where the drug effect is strong and the amount of drug required is small, such as vaccines [23,29]. Zeng et al. [30] used MN arrays coated with immune-polyelectrolyte multilayers to expand melanoma-specific T cells in vivo. The coating itself affects the sharpness of the needle, and friction may cause some of the drug to remain on the skin during penetration. Methods for enhancing the stability of the drug coating and reducing the loss rate of the drug under the premise of ensuring the efficient release of the drug require more research in the future. In addition, it causes waste, as the used needles need to be discarded.

### 2.3. Hollow MNs

With hollow MNs, the drug is preloaded into the needle body with a hole in the tip and then injected into the skin using a syringe or micropump to pressurize it (Figure 1C). Among the various MN types, hollow MNs have the largest amount of one-time infusion and the most accurate dosage, and the speed can be freely adjusted (similar to injection). However, the manufacturing process of hollow MNs is precise and the manufacturing cost is high. The disadvantage is that the pinhole is easily blocked by the skin tissue, and the angle of the needle wall is not designed properly, which can lead to the drug spilling out of the skin during injection; furthermore, the physical strength of the needle is low, and it can easily break and remain in the skin [31,32].

### 2.4. Soluble or Degradable MNs

Soluble or degradable MNs are a kind of MN that is being studied a lot at present. The MN body can be made of biodegradable or water-soluble polymers (polylactic acid, polycarbonate, polyglycolic acid, polylactide ethyl lactide, etc.). After entering the skin, the needle body is degraded or dissolved within minutes or hours, whereby the needle body separates from the substrate and releases the internal drug after degradation or dissolution [29]. The molecules are encapsulated in MNs, which can be dissolved in the skin for bolus or sustained delivery without leaving biohazardous sharp medical waste (Figure 1D) [25,33,34]. Soluble MNs with different degradation rates can be prepared by changing the material and shape of the needle body according to the treatment requirements. Since they are not used repeatedly, this method reduces the risk of cross-infection, and medical waste is more easily disposed of without sharp waste left after use.

### 2.5. Swellable MNs

Swellable MNs are a relatively new kind of MN, and are prepared from a cross-linked hydrogel and can expand but not dissolve after absorbing interstitial fluid (IF). This kind of hydrogel MNs can be loaded in two ways: one is to pre-position the drug at the base of the MNs, and after piercing into the skin, the hydrogel absorbs the intercellular fluid to expand, forming a gel channel [32]. The other is that the hydrogel MN substrate and the needle body are both prepared by a mixture of drugs and polymers [26]. After penetrating the skin, the fluid penetrates, the needle swells, and the drug is released (Figure 1E). In addition, the application of swellable MNs is not limited to drug delivery, but can also be used to extract IF for subsequent analysis [35].

### 2.6. Porous MNs

Porous MNs have recently been researched owing to their distinctive and unique characteristics, where porous structures inside MNs with continuous nano- or micro-sized pores can transport drugs or biofluids by capillary action [21]. However, they are inherently fragile, due to the large number of pores and require hard inorganic materials to make porous MNs [36,37,38]. As a result, porous MNs have received relatively little attention in the past due to their complex manufacturing process and the ease with which they fracture. In recent years, porous MNs have shown promise for drug/vaccine delivery and ISF extraction/biosensing. In addition, as low-invasive partial breaking of the stratum corneum and the wholly interconnected micropores, porous MNs have been successfully applied for efficient drug delivery (penetration) and analysis of ISF (extraction) (Figure 1F) [27,39].

## 3. Fabrication of MNs

### 3.1. Materials and Properties of MNs

Compared with traditional syringe and needle transdermal delivery, the requirements of MNs are higher in terms of material selection and design, depending on its purpose. The materials from which MNs are constructed mainly include silicon, glass, ceramic, metal, hydrogel, polymer, sugar, etc. Common matrix materials for degradable or soluble MNs include sodium carboxymethyl cellulose (CMC), polyvinylpyrrolidone (PVP), hyaluronic acid (HA), polylactic acid (PLA), chitosan, etc. Advanced biodegradable metals have excellent biocompatibility and sufficient mechanical properties, and are also a potential kind of matrix material [40,41]. The design materials and characteristics of MNs vary greatly according to the application and administration requirements.

Ideal products must meet the requirements of good mechanical performance and insertion performance, which are closely linked to their materials and design. The transdermal properties of some typical recent MNs are listed in Table 1.

### 3.2. Design of MNs

MNs have been investigated for decades; in this review, the construction idea of MNs is divided into two main categories on the basis of MN design concept with respect to their precursors: either the substrate material is formed by physically mixed stacking casting [54], or by forming chemical bonds between various materials to increase the strength of the MNs [33]. It can be a single material [55] or a multilayer combination [16]. Physical mixing is relatively traditional, and is the most common method of MN preparation. With the deepening of materials science research, the design of base materials is becoming increasingly diverse. The most promising approach is to design functional and targeted materials to meet the demands of specific diseases or biological microenvironments. The earliest solid MNs break the skin corneum layer by puncturing the skin to produce channels, with most using non-biodegradable materials such as silicon and metal, with the MNs molded from a single material. However, nowadays, an increasing number of biologically safe materials that are soluble or biodegradable are being used to build MNs with multilayer structures but different properties in drug delivery. For example, an MN patch was designed with effervescent backing, and when the MN patch was applied on the skin, MNs were rapidly delivered through the fast dissolution of the effervescent backing [16].

### 3.3. Fabrication Technology of MNs

**Figure 2:**
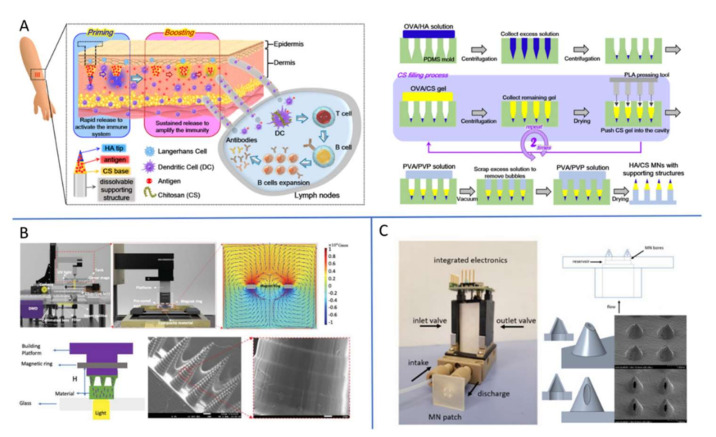
(**A**) The fabrication process for the HA/Chitosan MNs with polyvinyl alcohol/polyvinyl pyrrolidone (PVA/PVP) supporting structures. OVA: ovalbumin; PLA: poly(L-lactide-co-D, L-lactide). Adapted with permission from Ref. [56]. (**B**) Limpet tooth-inspired painless microneedles fabricated by magnetic field-assisted 3D printing. Adapted with permission from Ref. [61]. (**C**) A novel 3D-printed hollow microneedle microelectromechanical system. Adapted with permission from Ref. [60].

Manufacturing technology is one of the important factors affecting the development and application of MN transdermal drug delivery technology. Based on the substrate material and the characteristics of the loaded drug (such as the stability of the sensitivity to temperature and light), different manufacturing processes need to be selected. In general, MN production can take place through either a mold-based process or a mold-free process. The mold-based process is currently the simplest and most popular one. A mold made by copying the metal or stainless-steel master structures is required first, and the mold usually used is polydimethylsiloxane (PDMS) mold [29]. Typical MNs and mold fabrication techniques are listed Table 2. MN masters are usually produced using these techniques. Then, after mold fabrication, microneedles can be replicated on it. Here, as presented in Figure 2A, composite MNs consisting of a sodium hyaluronate tip and chitosan were developed using this mold-based approach for the rapid and continuous release of bipolar antigen [56]. Among mold-free technologies, mainly for polymer MN fabrication, additive manufacturing, or 3D printing, has proved to be an emerging field in MN structure fabrication. Three-dimensional (3D) printing is a manufacturing process for constructing objects from computer-aided design models [57,58]. In the field of drug delivery, 3D printing has already gained visibility and has been recognized by the respective regulatory bodies (e.g., FDA) [59,60]. Compared to other microscale manufacturing methods, 3D printing easily overcomes challenges in the fabrication of MNs with complex geometric shapes and multifunctional performance. As shown in Figure 2B,C, a magnetic-field-assisted 3D printing (MF-3DP) process has been used to build MNs, using aligned iron oxide nanoparticles (aIOs) and polymer matrix materials, as well as a novel 3D-printed hollow microneedle microelectromechanical system combining two cutting-edge technologies—3D printing of MNs and Microelectromechanical System (MEMS)—to create a 3DMNMEMs device [60,61]. However, it remains a challenge to use 3D printing technologies to fabricate microscale MNs with fine detail that meets the necessary degree of precision and strong mechanical performance; this is, therefore, a bottleneck that hinders application in the clinic. To date, researchers in this field have developed and optimized many MN fabrication methods. In practice, the complete process route for making target MNs usually requires a combination of multiple techniques [62].

## 4. Biomedical Applications and Latest Research Progress of MNs

**Figure 3:**
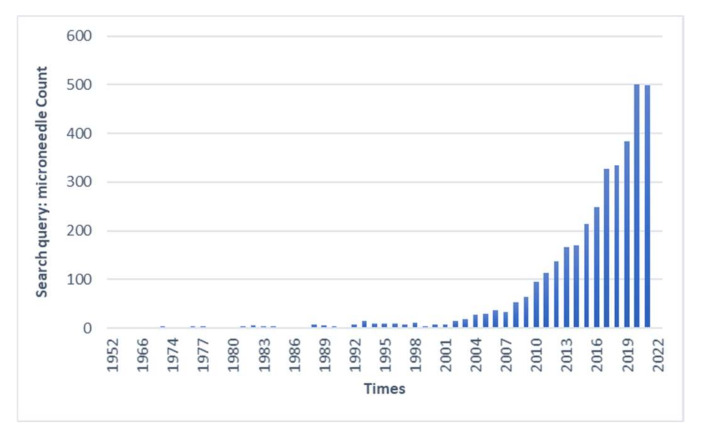
Statistical analysis of previously published papers obtained by searching the keyword “microneedle” on PubMed.

With the rapid development of manufacturing technology, the application of MNs has been extended to many fields, such as immunobiology, disease diagnosis, and cosmetology. By searching the keyword “microneedle” on PubMed, 3,179 results were retrieved (as shown in Figure 3). Among these, the earliest report on MNs was in 1952, when REAUME of Yale University proposed the use of hydrofluoric acid in making glass MNs [89]. The early MNs emerged as a useful tool mainly for laboratory research in fields such as experimental embryology, tissue culture, and electron microscopy [90,91,92,93,94,95]. Applications of MNs increased significantly in the 1980s, but were largely limited to the delivery of proteins, DNA, and more in the field of laboratory cell engineering [96,97]. It is said that MNs were first proposed in 1976, and a US patent for transdermal MNs administration was issued simultaneously [11,20]. Although MNs were first proposed in the 1970s, the technology for making micron-sized needles was not widely used until a little over two decades ago. MNs have attracted wide attention in the field of medicine. The manufacture and design of MNs have been studied and innovated continuously, and they have entered the clinical trial stage [98].

### 4.1. The Treatment of Osteoarthritis

Osteoarthritis (OA) is the most common degenerative joint disease, affecting more than 250 million people worldwide [99]. Since osteoarthritis is the most common type of arthritis and is a major cause of disability, there is a great need for disease treatment and symptomatic treatment [100]. However, the current treatment methods are limited, and there is still a lack of effective treatment methods for OA. MN percutaneous administration, as a non-invasive route of administration, is an attractive alternative to parenteral administration. At the same time, percutaneous administration can avoid the enzyme degradation caused by the gastrointestinal tract and liver. The use of MNs offers several potential advantages for the treatment of OA, including avoidance of liver metabolism, reduction of the severe adverse reactions of oral drugs to the gastrointestinal tract, achievement of long-term and sustained drug release, and ease of terminating drug delivery in the event of toxicity.

**Figure 4:**
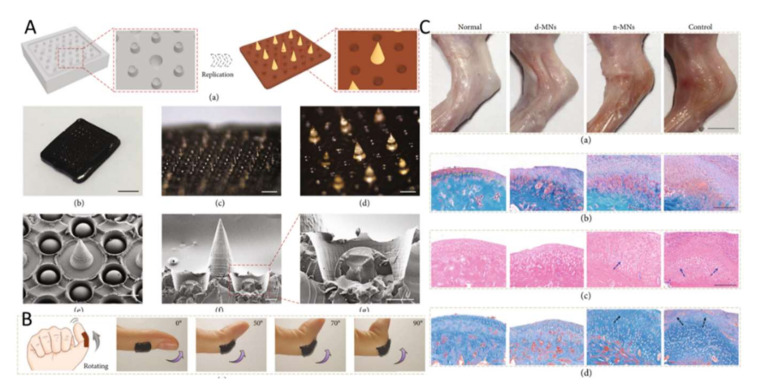
MNs loaded with glucocorticoid were used to treat knee osteoarthritis in rats. (**A**) Fabrication and characterization of the bioinspired multifunctional MNs. (**B**) Measurement of adhesion ability. (**C**) Evaluation of the multifunctional MNs on treatment for the KOA rat model. Adapted with from Ref. [54].

Multi-functional bionic MNs allow drugs to be delivered to parts of the body with high flexibility sustainably and over a long period. After MNs loaded with glucocorticoid were used to treat knee osteoarthritis in rats, the degree of swelling and inflammation in the knee joints of rats was greatly reduced (as shown in Figure 4) [54]. Meloxicam is a drug with poor water solubility. As with other NSAIDs, the occurrence of gastrointestinal side effects results in poor patient compliance. In addition, because OA is a chronic disease, drugs for treating arthritis are often taken for a long time. Amodwala et al. used polyvinyl alcohol and polyvinyl alcohol pyrrolidone to encapsulate meloxicam with MNs, and in vivo pharmacodynamics experiments in rats showed anti-inflammatory activity comparable to that of approved oral tablets [18]. The safety, high efficiency, and stability of the MN patch were proved. At the same time, Chen et al. designed rapidly produced two-layer MNs, composed of a soluble needle body and an insoluble substrate, and using water-soluble polymer to encapsulate meloxicam at the tip of the MNs [14]. The entire process of making the MNs took only about an hour. The results showed that the MNs had the advantages of rapid drug release (91.72% within 30 min), effective skin administration (79.18%), no obvious skin irritation, good relative bioavailability (122.3%), strong anti-inflammatory and analgesic effects, etc. As a transdermal drug delivery approach, MNs are able to solve the problems of the low oral utilization rate of some drugs and poor patient compliance. However, few MNs have been reported for the treatment of osteoarthritis.

### 4.2. The Treatment of Rheumatoid Arthritis

**Figure 5:**
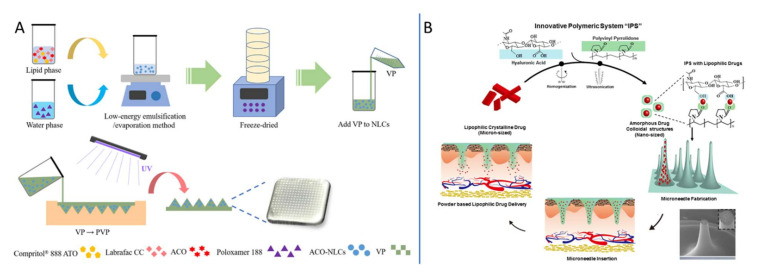
Nanocarriers combined with MNs for drug delivery to treat rheumatoid arthritis (RA). (**A**) Schematic representation for the ACO-NLCs-loaded dissolving MNs (ACO-NLCs-MNs) fabrication procedure. Adapted with permission from Ref. [107]. (**B**) Schematic representation for innovative polymeric system (IPS) for solvent-free lipophilic drug transdermal delivery via dissolving MNs, and the Scanning Electron Microscopy (SEM) image. Adapted with permission from Ref. [108].

In addition to meloxicam used for OA, some other drugs have also been combined with MNs technology for the treatment of rheumatoid arthritis, such as methotrexate [101,102,103], artemether [104,105], alkaloids [106,107], capsaicin [108], neurotoxin [109], triptolide [110], paeoniflorin [111], sinomenine [112] and Etanercept (EN) [113]. MN transdermal delivery thus offers a promising route of administration for arthritis. It is used to control the release rate, reduce the dosage, and avoid the risk of drug side effects with minimally invasive drug delivery. It is noteworthy that the nano-lipid carrier was used as the drug carrier, and the drug was encapsulated with the carrier until it was delivered by soluble MNs, successfully solving the limitation of drug solubility [107]. As shown in Figure 5A, the nanostructured lipid carriers (NLCs) eliminated the hepatic first-pass metabolism and toxicity, while MNs technology significantly enhanced drug delivery, forming cutaneous depots to promote the sustained release of the drug [107]. In addition, Dangol et al. [108] studied innovative polymeric system (IPS) for solvent-free powered lipophilic drug transdermal delivery via dissolving MNs (DMNs). The IPS facilitated phase transition of micron-sized crystal lipophilic drug into a transdermal diffusible nano-sized amorphous form and fabricated the DMN backbone for transdermal delivery, as shown in Figure 5B. The results demonstrated that the IPS-based DMN with powder capsaicin was a therapeutically efficient means of inflammation treatment in rheumatic arthritis.

### 4.3. The Treatment of Dermatology Dermatosis 

The skin is an important line of defense in the human body, and skin-related diseases are one of the main research directions of MNs at present. After skin injury, mesh with the function of avoiding wound infection and promoting tissue remodeling has important value in wound healing. Typical patches have the characteristics of simple microstructure and slow drug delivery, which limits the repair effect and further medical application of the patch. Yet MNs have porous microstructures and could use antibacterial multifunctional polymer materials to encapsulate skin growth factor or drugs to achieve sustained release or controlled release. For example, chitosan (CS) is a unique alkaline polysaccharide in nature that has prominent characteristics of biodegradation, non-toxicity, biocompatibility, natural antibacterial, hemostasis, etc., and is widely used to develop multi-functional smart micron array (CSMNA) patches for wound healing [48]. Furthermore, Yang et al. [19] combined the drug inducing hair follicle activation with exosomes derived from mesenchymal stem cells (MSC) and small-molecule drug UK5099 in the MN patch for drug administration, and selected keratin, a natural protein derived from hair, as the matrix material for MNs to regulate hair follicle cycle transition and promote hair regeneration. The MN combined drug delivery method not only overcomes the disadvantage of low transdermal efficiency when the drug is applied externally by UK5099, but also solves the problem of short drug residence time and frequent injection when the exosome is administered by subcutaneous injection.

### 4.4. The Delivery of Vaccine

MNs used in vaccine delivery comprise an earlier field of study, making it a more mature subject. In particular, MN vaccine can be stored at room temperature and transported in solid form, and can be used for the packaging or encapsulation of DNA vaccine, subunit antigen, or inactivated or live virus vaccine [114]. It also offers a painless, non-invasive, convenient method for the management and transmission of reagents, without requiring a cold chain for storage and transport, and reducing sharp medical waste. In addition, it can reduce damage and the spread of infectious blood-borne diseases in developing countries or rural areas without good medical conditions and management [115]. Recently, coronaviruses have posed a serious threat to global health, and there is an urgent need for a safe vaccine that can quickly produce an effective and long-lasting virus-specific immune response against these pathogens. In the study of Kim et al. [116], the researchers delivered MERS-CoV vaccine and SARS-CoV-2 vaccine to mice by routine subcutaneous needle injection or by percutaneous administration of dissolved MN arrays (MNA), and performed a comprehensive preclinical immunogenicity analysis of the MERS-CoV vaccine in mice. The mice produced anti-SARS-CoV-2 antibodies within two weeks. Mice immunized with the MERS-CoV vaccine produced antibody levels sufficient to neutralize the virus for at least a year, and those immunized for SARS-CoV-2 seemed to follow the same trend. In addition, MNA delivery of these vaccines was shown to produce a stronger immune response than traditional hypodermic needle injections. This suggests that MNA vaccination is a promising immunization strategy for coronavirus infection. Importantly, the novel coronavirus MN vaccine remains effective even after being thoroughly sterilized with gamma rays. This is a critical step in the development of a vaccine suitable for humans. MNA delivery has the potential to speed up the vaccine production process and significantly reduce costs by reducing the required vaccine dose, and the development and advances in MNs technology are expected to enable faster responses to emerging pandemics [117].

### 4.5. The Treatment of Cancer

MNs have been employed to deliver anticancer drugs (chemotherapy), as well as photodynamic therapy (PDT) and photothermal therapy (PTT) agents, to skin tumor sites in a minimally invasive fashion and with improved therapeutic efficacy. In mice with subcutaneous melanoma tumors, the delivery of ovalbumin-pulsed dendritic cells via cryomicroneedles elicited higher antigen-specific immune responses and led to slower tumor growth than intravenous and subcutaneous injections of the cells. Cryomicroneedles used for delivery cells could retain their viability and proliferative capability. Biocompatible cryomicroneedles may facilitate minimally invasive cell delivery for a range of cell therapies [118]. The combination of MN percutaneous delivery with cancer immunotherapy to improve the efficacy of cancer treatment is also an attractive direction. Duong et al. [119] proposed soluble MN combination delivery for the release of vaccines and adjuvants in skin tissues. Compared with subcutaneous injection of the MN-free nanocomposite, subepithelial implantation of MNs combination induced stronger antigen-specific antibody response. Compared with traditional vaccination, the soluble MN combination enhanced the antibodies’ memory. The combination of photothermal therapy and chemotherapy has great potential to improve the efficacy of cancer treatment and even promote its eradication, preventing cancer recurrence caused by residual cancer cells [46,51]. Moreira et al. [51] prepared layered polyvinylpyrrolidone microneedles coated with chitosan loaded with adriamycin and polyvinyl alcohol rich in AuMSS to mediate the transmission of adriamycin and AuMSS nanorod (Dox@MicroN) to cancer cells. The Dox@MicroN patch was proved to be a simple large-scale delivery device, and improved the therapeutic effect of tumors in combination with chemotherapy–photothermal therapy. This microneedle delivery system can be used to mediate the local delivery of the new drug-photothermal combination, avoiding all the problems associated with the systemic delivery of anticancer drugs.

### 4.6. Usage in the Contraception Field

**Figure 6:**
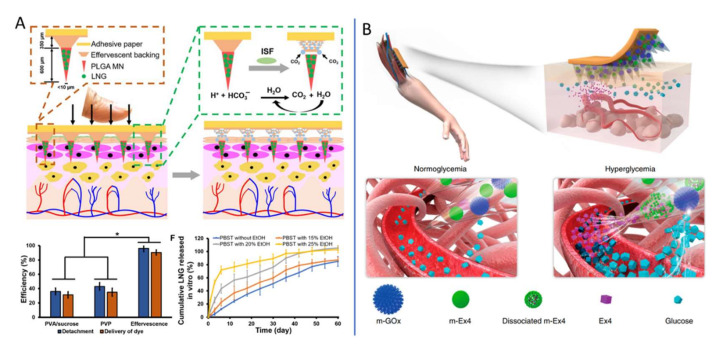
MNs are used in other biomedical applications. (**A**) Long-acting reversible contraception by effervescent MN patch. Adapted from Ref. [16]. (**B**) Glucose-responsive Ex4 delivery MN-array patches. Adapted with permission from Ref. [121].

Since MN patch technology was proposed, it has been applied in many biomedical fields, showing great application prospects. Li et al. [16] extended the application of the MN patch to the field of contraception. Effective contraception is important for women’s health. To increase long-term contraception, they developed a long-acting contraceptive MN patch, which is easy to use and does not produce harmful sharp waste. As shown in Figure 6A, biodegradable polymer PLGA was loaded with contraceptive hormone (levonorgestrel, LNG) to form a microneedle body that releases LNG over a duration of about 1 month through the slow degradation of PLGA in the body. These studies showed that effervescent microneedle patches can promote greater long-acting contraception [120].

### 4.7. The Treatment of Diabetes

Insulin is a natural hormone secreted by pancreatic islet cells in the pancreas to help the body regulate blood sugar. Long-term insulin injections are often necessary for diabetics, whether they are type 1 (which cannot produce insulin itself) or type 2 (which cannot respond to normal levels of insulin). However, there is difficulty in controlling blood sugar with insulin. When the dose is wrong, there may be a risk of hypoglycemia, which in severe cases can lead to seizures, coma, and even death. As a result, diabetics need to constantly monitor their blood sugar levels while taking insulin [122]. Insulin delivery by MNs is one of the most advanced and important directions of MN application. In 2015, Zhen Gu’s research group first reported the concept and prototype of the “Smart Insulin Patch”, and founded the Zenomics company to promote its clinical transformation. The basic idea is to combine a preparation that responds to blood sugar to release insulin with an MN array made of a polymer material (HA). The use of MNs less than 1 mm in size can reduce the pain caused by injection and improve the quality of life of patients [123]. The team continued to improve the technology of the new “smart insulin patch” for blood glucose response and has completed several stages of progress [50,122,124]. In addition to responding to glucose, as shown in Figure 6B, Chen et al. [121] combined m-GOx and m-Ex4 to design a smart pH-responsive Ex4 MNs delivery patch based on alginate for the treatment of type 2 diabetes. Metformin as a hypoglycemic agent is also embedded in separable MNs composed of polyvinyl alcohol and sucrose (PVA/Suc), which is released as needed under NIR irradiation [125].

### 4.8. Other Applications

Initially, MNs were used to break barriers and enhance transdermal efficiency. Now, the use of microneedles has expanded to many other fields. The prominent studies of biomedical application of MNs were list in Table 3. In addition to the treatment or drug delivery of various diseases, as described above, in the skin, MNs have also been used to treat the eye, cells, blood vessels, and so on [2]. In particular, hollow microneedles and porous microneedles are mostly used in biosensor research, and good results have been obtained using them. Takeuchi et al. [36] interfaced porous MNs with a microfluidic chip consisting of a capillary pump for the continuous sampling of ISF, which will lead to minimally invasive and continuous biosampling for long-term healthcare monitoring. With increasing crossover between disciplines, microneedles will be able to take on more functions. Chen et al. combined physical (MNs) and nonphysical (enhancer) modes of drug delivery enhancement for a macromolecule in a large animal model [126]. These systems could potentially enable the delivery of a range of drugs through the generation and maintenance of a privileged region in the gastrointestinal tract.

MN drug delivery technology is a new transdermal drug delivery method based on the dual drug release characteristics of subcutaneous injection and transdermal patch technology. Research into the drug treatment of chronic illnesses such as arthritis or diabetes, cancer, dermatocosmetology, family planning, genes, and epidemic disease prevention demonstrates a diversification of development, including the use of small molecule chemistry medicine, biological macromolecule polypeptides, protein and gene medicine, insulin, and so on. Local or systemic administration is also carried out according to disease type and drug performance, and drugs with long-term or short-term release have been designed for use with MNs.

## 5. Outlook: Nanotechnology Potentiates MNs in Biomedicine

On the basis of the previous studies, it can be seen that the functional design of MN drug delivery systems using nano-engineering technology could make it possible to realize intelligent control of drug release amount, release kinetics, or stimuli-responsive delivery. The main advantage of nanomaterials is that they can be designed for specific functions, and are responsive to specific microenvironments [5]. There are special microenvironments and landmark substances at the lesion site that could be extensively harnessed for the design of functional nanomaterials incorporating MNs for different diseases therapy. It has been reported that high-density avascular chondrocyte extracellular matrix (ECM) impeded drug penetration, but nanoparticles (NPs) (<96 nm) can enter the dense ECM to extend drug retention [127,128,129]. These characteristics provide a starting point for the design of functional nanomaterials. For example, the upregulation of MMP-13 is a key step in OA pathogenesis, leading to the degradation of cartilage ECM. Chen et al. [127] designed novel biocompatibility targeting cartilage and MMP-13/pH response ferritin nanocage (CMFn) containing an anti-inflammatory drug (Hydroxychloroquine, HCQ) for OA imaging and treatment. CMFn@HCQ nanocages can target cartilage and continuously release HCQ in OA joints under acidic pH conditions, thereby extending drug retention time to 14 days and significantly reducing synovitis in OA joints.

Since the emergence of MNs as a promising drug delivery technology, MNs have developed into a broad and rapidly growing research field [10,114]. The rapid development of nanotechnology and materials science has provided a great boost to the research and innovation of MNs, providing new ideas for the treatment of disease and better treatment possibilities. Nanotechnology can be used for engineering biomaterials or drugs to integrate more functions that can achieve intelligent drug delivery in the targeted lesion site. MNs are a very suitable carrier for bioactive nanoparticles. Combining nanotechnology with MNs technology could bring new hope for the treatment of patients.

## Figures and Tables

**Table 1:** Transdermal properties of MN composites.

Materials	Application	Morphology	Mechanical Strength	Insertion Capability	Reference
PVP, PVA	Bacterial biofilm skin infection	Needle density of 16 × 16, pyramidal needles; 850 μm height [600 μm pyramidal tip, 250 μm base column] and 300 μm width at the base and 300 μm interspacing	After the application of 32 N/MN array, the reductions in MN height were found to be 12.36 ± 3.12%, 13.03 ± 2.71%, 12.65 ± 3.22%, 12.98 ± 2.09%, 13.16 ± 3.10%, 12.91 ± 2.98% and 13.21 ± 2.11%, respectively. The percentage of height reduction of MNs after the application of 32 N/MN arrays, equivalent to human manual compression pressure [42].	In the full-thickness porcine skin, the penetration depth of 503.65 ± 12.43 μm, 500.43 ± 10.12 μm, 506.43 ± 21.11 μm, 498.43 ± 10.41 μm, 502.11 ± 10.03 μm and 499.87 ± 10.03 μm.	2020 [43]
PVP (30,000 Da), CMC (250,000 Da), HA (200,000–300,000 Da)	Melanoma	10 × 10 square pyramidal MNs, and geometry, that is, 350 μm base width, 700 μm height, about 15 μm tip width, and 500 μm needle center-to-center spacing	Transdermal applications (threshold, 0.15 N). Go (500 μg/mL) increasing CMC failure force the up to about 0.2 N	Complete insertion (98–100%) of PVP MNs by incorporating 500 μg/mL GO	2020 [44]
PVP(K29/32)	Immune	An 8 mm × 8 mm array, and uniformly distributed with 225 (15 × 15) square pyramid needles having a bottom edge of 200 μm and a height of 600 μm		The depths of the holes created by MN needles were about 150 μm.	2020 [45]
PVP(10 kDa)	Anti-angiogenesis, skin tumors and vascular anomalies (vas)	Had a uniform structure of pyramidal shape and contained 100 needles (i.e., a 10 × 10 array) with a length of 800 ± 15 μm.	The compression force increased to 0.6 N/needle when the displacement increased to 0.6 mm.	Deliver RAPA to a depth of 200 μm.	2020 [46]
resin	chronic wounds	With different needle spacings (1.5–3 mm), needle lengths (0.8–3 mm), base sizes (0.5–1.5 mm), and opening diameters (0.2–0.5 mm). a length of about 2 mm was selected	Mechanical tester: the MNAs did not break and were only bent under a compressive force of about 78 N. Pigskin: no deformation or breakage was observed upon penetration and removal from the pigskin.	The majority of the MNAs penetrated fresh pigskin with less than 2N of force and full penetration was achieved with about 7N.	2020 [47]
Chitosan (180 kDa)	Wound healing	A 20 × 20 mn array and each MN possessed a conical shape with a tip diameter of 5 μm, a height of 600 μm, and a base diameter of 300 μm			2020 [48]
PVA (Mw: 31,000–50,000, 87–89% hydrolyzed)	Diabetes	15 × 15 array, 650 μm needle length, the total area of 11 × 11 mm^2^. A base of 315 μm and a height of 650 μm	0.71 N/needle, which is efficient for skin penetration		2020 [49]
N-vinylpyrrolidone (NVP)	Insulin	a 20 × 20 array; each needle had a pyramidal shape, with a width of 400 µm at the base and a height of 900 µm	The fracture force of the MNs to be 0.90 ± 0.35 N/per needle using a tensile compression machine	The MN patch could effectively penetrate the skin of the minipig.	2020 [50]
PVA (Mw: 31,000 g/mol), PVP (Mw: 360,000 g/mol), chitosan (Mw: 50,000–190,000 Da)	Cancer	A 16 (4 by 4) MN array, a bevel-like structure with 425 µm, 1420 µm, and 1740 µm of width, height, and tip-to-tip distance, respectively.			2020 [51]
resin	Anti-wrinkle	The MN illustrated here are that of MN height 800 μm; MN tip diameter 100 μm; MN base diameter of 400 μm and MN interspacing of 800 μm.	able to withstand breakage from a typical thumb force of about 20N	The approximate depth of penetration for the intact skin is about 220 μm; FMNP about 480 μm and PMNP about 750 μm.	2020 [52]
pullulan (viscosity: 133 mm^2^/s, 10% w/w, Ubbelohde type viscometer, average Mw 200 kDa)	Small molecule drugs and biomolecules	Displayed well-formed DMNs with sharp tips, a complete array of needles.	An insertion force of 0.089 N per needle for 30 s may be suitable for penetrating the skin	403 ± 35.8 μm inserted out of the total height of 504 ± 6.4 μm which is 80 ± 7.2% insertion.	2019 [53]
MeHA	Timely metabolic analysis	The obtained MN patch displayed a height of about 800 µm with a base width of about 250 µm and inter needle spacing of about 450 µm.	A thumb press (about 1.5 N) could easily penetrate the MNs into porcine skin.	The efficient penetration depth of MN was about 300 µm.	2017 [35]

PVP: poly(vinylpyrrolidone); PVA: polyvinyl alcohol, vinylalcohol polymer; CMC: carboxymethyl cellulose; HA: sodium hyaluronate; NVP: N-vinylpyrrolidone; MeHA: methacrylated hyaluronic acid.

**Table 2:** Typical MN fabrication techniques.

Fabrication Methods		Reference
Mold-free methods/master structures	Photolithography	2008 [63]
	Dry etch	1998 [64], 2010 [65]
	Wet etch	2010 [66]
	Laser cutting	2007 [67]
	Electroplating or electroless-plating	2011 [68]
	Micro milling and micro grinding	2018 [69], 2013 [70]
	Drawing lithography	2010 [71], 2013 [72]
	Electro-drawing	2018 [73], 2014 [55]
	Magnetorheological drawing lithography	2018 [74]
	Droplet-born air blowing (DAB) method	2013 [75]
	Lithography, electroplating, and molding (LIGA) technique	2007 [76], 2003 [77]
	Stereolithography (STL)	2018 [78]
	Fused deposition modeling (FDM)	2018 [79]
	Micro-stereolithographic 3D printing	2017 [80],2021 [60]
	A two-photon polymerization (TPP) 3D printing methodology	2019 [81]
	magnetic field-assisted 3D printing (MF-3DP)	2021 [61]
	Laser ablation and cutting	1997 [82]
Mold-based methods	Hot embossing	2018 [83]
	Injection molding	2018 [84], 2012 [85]
	Solvent casting	2012 [33], 2018 [56,86]
Coated method	Spraying	2014 [87]
	Dipping	2007 [88]
Method for forming porous structure	Electrochemical anodization	2022 [21]
	Wet etching	
	Mild micro-molding	
	Sintering process	
	Porogen leaching	
	Hot embossing	
	Phase separation	
	Emulsion and bonding	

**Table 3:** The prominent studies of biomedical application and latest research progress of MNs.

Biomedical Application	Latest Research
Osteoarthritis	Glucocorticoid [54], Meloxicam [14,18]
Rheumatoid arthritis	Methotrexate [101,102,103] Artemether [104,105] Alkaloids [106,107] Capsaicin [108] Neurotoxin [109] Triptolide [110] Paeoniflorin [111] Sinomenine [112] Etanercept (EN) [113]
Dermatosis	Exosomes and small molecule drug UK5099 and keratin [19]
Delivery of vaccine	MERS-CoV vaccine and SARS-CoV-2 vaccine [117]
Cancer	Delivery of ovalbumin-pulsed dendritic cells for subcutaneous melanoma tumors [119] Bioresorbable polypeptide matrix with a nanopolyplex, loaded ovalbumin-expressing plasmid OVA (pOVA) and immunostimulant-polyinosinic:polycytidylic acid (poly(I:C)), for B16/OVA melanoma tumors [120] Mediating the delivery of doxorubicin and aumss nanorods (Dox@micron) to cancer cells [51] Loaded rapamycin for skin tumors and vascular anomalies [51]
Contraception	Levonorgestrel [16]
Diabetics	Insulin delivery [50,122,123,124] Exendin-4 (Ex4) and glucose oxidase (GOx) delivery [125]
